# Supplementary material for: Tissue-distinct Features of Follicular Cytotoxic CD8+ T Cells in Trypanosoma cruzi infection
Source: bioRxiv. 2026 Feb 26:2026.02.25.707971. Preprint. [Version 1] doi: 10.64898/2026.02.25.707971 (PMC13160107; doi:10.64898/2026.02.25.707971)
Supplement: Supplement 1 — Figure S1. Transcriptional analysis of Tfc and non-Tfc cells from spleen and LN from T. cruzi infected mice. CXCR5+PD-1+ (Tfc) and CXCR5−PD-1− (non-Tfc) CD8+ T cells were isolated by cell sorting from spleen and inguinal LN of T. cruzi–infected mice at 18 dpi and subjected to bulk RNA sequencing. Principal component analysis of the whole transcriptome shows the distribution of Tfc and non-Tfc samples from both SLO. Each dot represents one biological replicate (N = 3), consisting of pooled cells from three mice. Table S1. Abs and reagents used for flow cytometry. Detailed list of Abs and reagents used in flow cytometry experiments, including their target specificity, fluorochrome conjugate, supplier, catalog number, clone, and working dilution. [file media-1.zip › supplementary material/Table S1.pdf]

Surface markers					
**Target**	**Fluorochrome/Biotin**	**Company**	**Catalog #**	**Clone**	**Dilution**
B220	FITC	Biolegend	103202	RA3-6B2	1/200
B220	PerCp-eFluor.710	eBioscience	46-0452-82	RA3-6B2	1/200
B220	PE-Cy7	eBioscience	25-0452-82	RA3-6B2	1/300
B220	BV421	Biolegend	103240	RA3-6B2	1/400
B220	APC-Cy7	Biolegend	103224	RA3-6B2	1/200
B220	BV711	Biolegend	103255	RA3-6B2	1/300
CD122	PE-Cy7	eBioscience	25-1222-80	TM-b1	1/100
CD127	BV711	Biolegend	135035	A7R34	1/100
CD138	APC	BD Biosciences	561705	281-2	1/200
CD138	BV421	BD Biosciences	562610	281-2	1/200
CXCR5	Biotin	BD Pharmingen	551960	2G8	1/75
ICOS	PerCp-eFluor.710	eBioscience	46-9942-82	7E.17G9	1/100
CD3e	PerCp/Cy5.5	eBioscience	45-0031-82	145-2c11	1/100
CD4	FITC	eBioscience	11-0041-85	GK1.5	1/200
CD4	PE-Cy7	eBioscience	25-0041-82	GK1.5	1/200
CD4	APC	Biolegend	100530	GK1.5	1/200
CD4	APC-eFluor 780	eBioscience	47-0041-82	GK1.5	1/200
CD4	PECF594	BD Biosciences	562285	RM4-5	1/300
CD40L	PE	BD Pharmingen	553658	MR1	1/100
CD44	PE-Cy7	Biolegend	103030	IM7	1/200
CD44	PE-Cy5	eBioscience	15-0441-81	IM7	1/200
CD62L	APC	eBioscience	17-0621-83	MEL-14	1/200
CD62L	SB 600	eBioscience	63-0621-82	MEL-14	1/200
CD8a	FITC	eBioscience	11-0081-86	53-6.7	1/200
CD8a	AlexaFluor 700	eBioscience	56-0081-82	53-6.7	1/200
CD8a	PE-Cy7	eBioscience	25-0081-82	53-6.7	1/300
FasL	PE	BD Biosciences	555293	MFL3	1/100
GLUT1	Biotin	Novus Biologicals	NB110-39113B	Polyclonal	1/100
H-2Kb/TSKB20	BV421	NIH Tetramer Core Facility	-	-	1/400
IgD	FITC	eBioscience	11-5993-82	11-26c	1/200
KLRG1	APC	Biolegend	138412	2F1/KLRG1	1/200
MCT1 (SLC16A1)	APC	eBioscience	AMT-011-APC50UL	Polyclonal	1/100
PD-1	PE-Cy7	eBioscience	25-9985-82	J43	1/100
PD-1	APC	eBioscience	17-9985-82	J43	1/100
PD-1	Brillant Violet 421	Biolegend	135221	29F.1A12	1/100

Secondary reagents					
**Reagent**	**Fluorochrome**	**Company**	**Catalog #**	**Clone**	**Dilution**
Goat anti-Rabbit IgG	Alexa Fluor 546	eBioscience	A-11071	Polyclonal	1/500
Streptavidin	PE	eBiosciences	12-4317-87	-	1/300
Streptavidin	PE-Cy7	eBioscience	25-4317-82	-	1/300
Streptavidin	APC	BD	554067	-	1/300

Intracellular / transcription factors / cytokines					
**Target**	**Fluorochrome**	**Company**	**Catalog #**	**Clone**	**Dilution**
Bcl-6	AlexaFluor 647	BD Bioscience	563582	K112-91	1/75
CD107a	PE	Biolegend	121612	1D4B	1/200
CD107a	APC/Cy7	eBioscience	121616	1D4B	1/150
Eomes	PE-eFluor610	eBioscience	61-4875-82	Dan11mag	1/400
Granzyme A	PerCp-eFluor.710	eBioscience	46-5831-80	GzA-3G8.5	1/200
Granzyme B	FITC	Biolegend	515403	GB11	1/100
IFNg	BV711	BD Bioscience	564336	XMG1.2	1/200
IL-21	APC	eBioscience	17-7211-82	FFA21	1/50
IL-10	PE	Biolegend	505008	Jes5-16E3	1/100
IL-6	eFluor450	eBioscience	48-7061-82	MP5-20F3	1/75
IL-2	BV421	Biolegend	503837	JES6-5H4	1/75
IRF4	PerCp-eFluor.710	eBioscience	46-9858-82	3E4	1/100
Perforin	APC	Biolegend	154304	S16009A	1/100
Phospho-Akt (Ser473)	PE	Cell Signaling	4060 s	D9E	1/100
Phospho-mTOR (Ser2448)	PerCp-eFluor.710	eBioscience	46-9718-42	MRRBY	1/100
Phospho-p70 S6 Kinase (Thr389)	-	Cell Signaling	9234T	108D2	1/75
T-bet	PE-Cy7	eBioscience	25-5825-82	4B10	1/100
TCF-1	PE	BD Bioscience	564217	S33-966	1/200
TNF	eFluor450	eBioscience	48-7321-82	MP6-XT22	1/200

Metabolic / functional probes			
**Reagent**	**Company**	**Catalog #**	**Concentration**
Mito Tracker Green FM	ThermoFisher Scientific	M-7514	100 nM
MitoTracker Orange CMTMRos	ThermoFisher Scientific	M-7510	100 nM
MitoStatus™ Red	BD	564697	100 nM
MitoSOX Red	ThermoFisher	M36008	5 uM
2-NBDG	ThermoFisher	N13195	10 nM

Viability Dyes			
**Reagent**	**Company**	**Catalog #**	**Dilution**
Live/Dead Fixable Aqua 405	eBioscience	L34966	1/400
Live/Dead NIR Fixable Viability Kit 633	eBioscience	L10119	1/800
CellEvent™ Caspase-3/7 Green	eBioscience	C10427	

Table S1
