# Supplementary figures and images for: Tissue-distinct Features of Follicular Cytotoxic CD8+ T Cells in Trypanosoma cruzi infection
Source: bioRxiv. 2026 Feb 26:2026.02.25.707971. Preprint. [Version 1] doi: 10.64898/2026.02.25.707971 (PMC13160107; doi:10.64898/2026.02.25.707971)

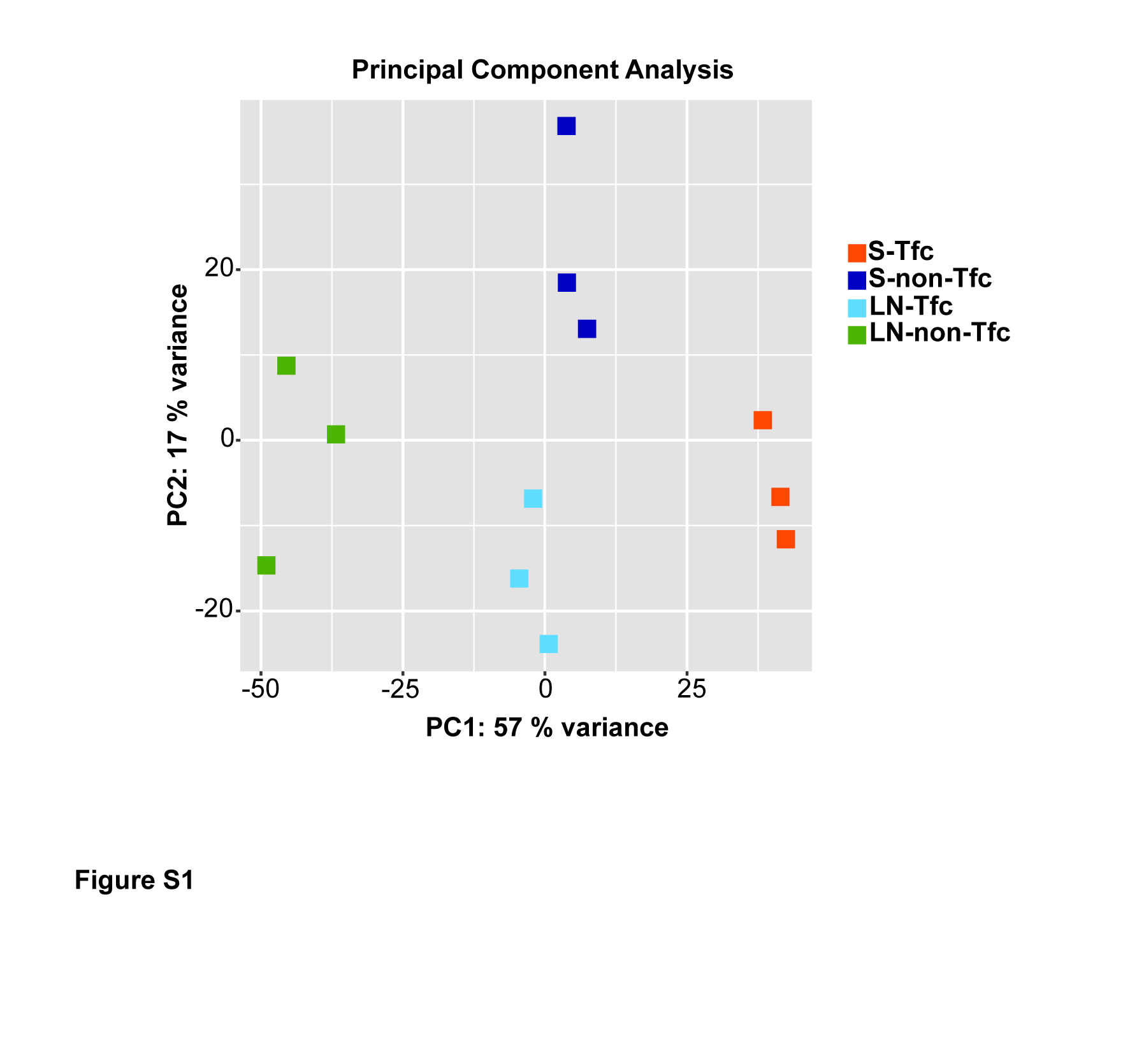

Supplement: Supplement 1 — Figure S1. Transcriptional analysis of Tfc and non-Tfc cells from spleen and LN from T. cruzi infected mice. CXCR5+PD-1+ (Tfc) and CXCR5−PD-1− (non-Tfc) CD8+ T cells were isolated by cell sorting from spleen and inguinal LN of T. cruzi–infected mice at 18 dpi and subjected to bulk RNA sequencing. Principal component analysis of the whole transcriptome shows the distribution of Tfc and non-Tfc samples from both SLO. Each dot represents one biological replicate (N = 3), consisting of pooled cells from three mice. Table S1. Abs and reagents used for flow cytometry. Detailed list of Abs and reagents used in flow cytometry experiments, including their target specificity, fluorochrome conjugate, supplier, catalog number, clone, and working dilution. [file media-1.zip › supplementary material/Figure S1.tif]
